# Pathogenic Variants of *SLC22A12* (URAT1) and *SLC2A9* (GLUT9) in Spanish Patients with Renal Hypouricemia: Founder Effect of *SLC2A9* Variant c.374C>T; p.(T125M)

**DOI:** 10.3390/ijms24098455

**Published:** 2023-05-08

**Authors:** Ana Perdomo-Ramirez, Elizabeth Cordoba-Lanus, Carmen Jane Trujillo-Frias, Carolina Gonzalez-Navasa, Elena Ramos-Trujillo, Maria Isabel Luis-Yanes, Victor Garcia-Nieto, Felix Claverie-Martin

**Affiliations:** 1 Unidad de Investigacion, Hospital Universitario Nuestra Señora de Candelaria, 38010 Santa Cruz de Tenerife, Spain; atter_rad@hotmail.com (A.P.-R.); acordoba@ull.edu.es (E.C.-L.); ctrufrix@gobiernodecanarias.org (C.J.T.-F.); cgonzaln@ull.edu.es (C.G.-N.); 2 Instituto Universitario de Enfermedades Tropicales y Salud Publica de Canarias (IUETSPC), Universidad de La Laguna, 38296 Santa Cruz de Tenerife, Spain; 3 Seccion Medicina, Departamento de Medicina Fisica y Farmacologia, Facultad de Ciencias de la Salud, Universidad de La Laguna, 38200 Santa Cruz de Tenerife, Spain; 4 Unidad de Nefrologia Pediatrica, Hospital Universitario Nuestra Señora de Candelaria, 38010 Santa Cruz de Tenerife, Spain; mabelyanes2@gmail.com (M.I.L.-Y.); vgarcianieto@gmail.com (V.G.-N.)

**Keywords:** renal hypouricemia, URAT1, GLUT9, renal tubular transport, pathogenic variant, haplotype analysis, founder mutation

## Abstract

Renal hypouricemia (RHUC) is a rare inherited disorder characterized by impaired urate reabsorption in the proximal tubule resulting in low urate serum levels and increased urate excretion. Some patients may present severe complications such as exercise-induced acute renal failure and nephrolithiasis. RHUC is caused by inactivating mutations in the *SLC22A12* (RHUC type 1) or *SLC2A9* (RHUC type 2) genes, which encode urate transporters URAT1 and GLUT9, respectively. In this study, our goal was to identify mutations associated with twenty-one new cases with RHUC through direct sequencing of *SLC22A12* and *SLC2A9* coding exons. Additionally, we carried out an SNPs-haplotype analysis to determine whether the rare *SLC2A9* variant c.374C>T; p.(T125M), which is recurrent in Spanish families with RHUC type 2, had a common-linked haplotype. Six intragenic informative SNPs were analyzed using PCR amplification from genomic DNA and direct sequencing. Our results showed that ten patients carried the *SLC22A12* mutation c.1400C>T; p.(T467M), ten presented the *SLC2A9* mutation c.374C>T, and one carried a new *SLC2A9* heterozygous mutation, c.593G>A; p.(R198H). Patients carrying the *SLC2A9* mutation c.374C>T share a common-linked haplotype, confirming that it emerged due to a founder effect.

## 1. Introduction

The levels of uric acid (UA), mainly found as urate at physiological pH in human serum, depend on the balance between hepatic production and degradation, reabsorption and secretion in renal proximal tubules, and secretion by the intestine [1]. The kidney plays a significant role in UA homeostasis because it reabsorbs, through the coordinated activity of several urate transporters expressed in the proximal tubule, around 90% of the UA filtered at the glomerulus [2]. Defects in two of these urate transporters cause renal hypouricemia (RHUC), an autosomal recessive renal disorder characterized by reduced reabsorption of UA in the proximal tubule (PT) and increased urinary urate secretion [3]. Although RHUC patients are frequently asymptomatic, some may present severe complications such as exercise-induced acute renal failure (EIARF) and nephrolithiasis [4,5,6,7,8,9]. The majority of RHUC cases have been detected by chance during routine health assessments, and a clinical diagnosis is reached using simple blood and urine tests [10]. Two types of renal hypouricemia can be distinguished based on the molecular alterations identified in the patients. Type 1 is characterized by loss-of-function mutations in the *SLC22A12* gene (RHUC type 1, MIM #220150) encoding urate transporter 1 (URAT1) [4,11]. URAT1 is a member of the organic anion transporter family required for effective urate reabsorption in the kidney [11]. This protein specifically localizes to the apical membrane of epithelial cells of proximal tubules. Inactivating mutations in *SLC2A9*, which encodes a voltage-dependent UA transporter known as glucose transporter 9 (GLUT9, a member of the GLUT family of hexose transporters) cause RHUC type 2 (MIM #612076). GLUT9 also plays an essential role in urate homeostasis [6,12,13,14,15]. Expression studies have shown that GLUT9 facilitates urate transport in exchange for both glucose and fructose [13,16]. GLUT9 contains 12 predicted transmembrane segments [17,18]. Two different isoforms, GLUT9L and GLUT9S, generated by alternative mRNA splicing of the *SLC2A9* gene and differing only at their amino-terminal cytoplasmic tails, have been described [17,19]. GLUT9L is encoded by 12 exons and contains 540 amino acids, while GLUT9S is encoded by 13 exons and is comprised of 512 amino acids. In the kidney, GLUT9L localizes to the basolateral membrane of proximal tubule cells, while GLUT9S is expressed at the apical membrane of collecting duct cells [19,20]. GLUT9-mediated urate transport has been shown to be voltage-dependent, an appropriate characteristic for urate efflux transport from the cell [12]. Based on information gained from some of these studies, Anzai and colleagues have proposed the following model of transcellular UA transport in the proximal tubule: URAT1 at the apical membrane reabsorbs UA from the lumen in exchange with intracellular monocarboxylate anions, while GLUT9 mediates the basolateral exit of the reabsorbed UA to the peri-tubular interstitium and the blood [12].

Homozygous or compound heterozygous mutations in *SLC22A12* cause most of the RHUC cases identified up to now. A total of 64 different *SLC22A12* mutations have been identified in patients with RHUC type 1, most of which (34) are missense mutations (Human Gene Mutation Database, HGMD. Available online: www.hgmd.cf.ac.uk). Functional studies have shown that some of these variants diminish the urate transport activity of URAT1 [4,11,21,22]. Initial reports of RHUC cases were from Japan and South Korea and showed that the *SLC22A12* variant c.774G>A; p.(W258X) is the predominant genetic cause of RHUC type 1 there [5,23]. Most likely, this mutation originated on the Asian continent and expanded in the Japanese population either by a founder effect or by genetic drift or both [24]. Subsequently, RHUC cases from Israel, several European countries, Pakistan, and China have been reported [6,7,9,22,25,26,27,28,29,30,31]. *SLC22A12* variants c.1245_1253del; p.(L415_G417del) and c.1400C>T; p.(T467M) are the main cause of RHUC type 1 in Roma populations of the Czech Republic, Slovakia, and Spain, and appear with very high frequency in these populations, 5.6% and 1.9%, respectively [9,22,32,33,34,35]. The Roma population, also known as ‘Gypsies’, constitutes the major native ethnic minority in Europe with an estimated population of approximately 11 million people, originating in India [36]. It is scattered throughout the continent and mainly concentrated in Central and South-Eastern Europe and the Iberian Peninsula [37,38]. Probably, founder effects and migrations have contributed to the high frequencies of variants c.1245_1253del and c.1400C>T in the Roma population.

Only 34 *SLC2A9* mutations (14 missense) have been identified in patients with RHUC type 2 (HGMD. Available online: www.hgmd.cf.ac.uk, accessed on 7 March 2023). Functional studies have shown that most of them decrease UA transport [6,14,25,39,40]. By screening 17 RHUC patients of 13 families from different regions of Spain, we have recently identified mutations in *SLC22A12* and *SLC2A9* [9,33]. A very rare *SLC2A9* mutation, c.374C>T, p.(T125M), has been detected in four of these families [9,33]. This variant was first identified in homozygosis in an Israeli RHUC patient of Sephardi-Jewish origin, and it has been shown to reduce the ability of GLUT9 to transport UA [25]. In the present study, we analyzed 21 new RHUC patients from 18 Spanish families and, notably, found 10 more patients (from 9 families) with variant c.374C>T. The detection of this recurrent mutation suggests the hypothesis of a founder effect. Therefore, we carried out an SNPs-haplotype analysis to determine whether patients with the c.374C>T mutation had a common-linked haplotype.

## 2. Results

### 2.1. Clinical Characteristics of Patients

All patients presented low levels of UA in serum (0.12 to 1.90 mg/mL) and increased urinary UA secretion (FE UA 12 to 190%) (Table 1). The levels of phosphate, glucose, and potassium were normal. In most cases, hypouricemia was detected incidentally. Renal function was normal in all patients. Fourteen patients were clinically asymptomatic, but seven presented renal symptoms including repetitive urinary tract infections, nephrolithiasis, rapid progressive glomerulonephritis, nephrotic syndrome, and scarring nephropathy (Table 1). We should also note that patient 38 (family 29) presented delayed psychomotor development. Patient 23 (family 16) presented recurrent urinary tract infections without nephrolithiasis, while his mother, patient 24, had recurrent nephrolithiasis. This family was of Roma origin and presented consanguinity (the father’s father and the mother’s father were first cousins). There was paternal consanguinity also in family 18. Patient 27 (family 19) was a 13-year-old boy who presented an idiopathic nephrotic syndrome. Subsequent analysis after corticosteroids treatment showed that proteinuria was negative. Patient P30 (family 21) was a 55-year-old woman of Roma origin who was admitted to the hospital for severe acute kidney injury due to rapid progressive glomerulonephritis (RPGN) associated with the positive anti-neutrophil cytoplasmic antibody (ANCA). At the time of the onset of acute kidney injury, the peak in renal function deterioration due to the glomerulonephritis was creatinine 4.87 mg/dL, and UA 6.5 mg/dL. Under immunosuppressive treatment the renal function improved. Once the renal function was normalized hypouricemia was evident (Table 1), with no alteration in phosphate, glucose, or potassium levels. UA determinations previous to the onset of acute kidney injury were lacking. Patient 31 (family 22), an 18-year-old boy of Roma origin, presented with acute kidney failure after intense exercise (EIARF) due to competition-level boxing training with a peak in creatinine at 5.7 mg/dL. There was acute tubular necrosis with preserved diuresis. After improvement in the renal function, elevated fractional excretion of UA was observed (Table 1). Previous analysis available showed that the patient had low serum levels of UA with normal creatinine levels (Table 1). Patient P40 (family 31) had a six-year-old brother with normal serum UA levels, although P40’s brother had increased urinary UA secretion (16%). Unfortunately, more data on the patient’s brother were not available.

We obtained clinical and biochemical data of patients’ relatives (mothers, fathers, sisters, or brothers) in six families (F15, F16, F19, F24, F26, F29, and F31). All of them were clinically asymptomatic, and in most of them the levels of UA in serum and urinary UA secretion were normal. However, the two brothers of patient 23 (family 16) presented low serum levels of UA (1.20 mg/dL and 1.00 mg/dL, respectively) and increased urinary UA secretion (FE UA 23% and 54%, respectively). The serum level of UA in their sister was normal.

### 2.2. Genetic Variants Identified in Patients with RHUC

Mutation analysis of the *SLC22A12* and *SLC2A9* genes using Polymerase Chain Rection (PCR) amplification and direct sequencing revealed *SLC22A12* variants in 10 patients and *SLC2A9* variants in 11 patients (Table 1). All patients of Roma origin presented the pathogenic *SLC22A12* variant c.1400C>T; p.(T467M) in the homozygous state, except one who had this variant in the heterozygous state together with the heterozygous *SLC22A12* deletion c.1245_1253del; p.(L415_G417del). In eleven families, we showed that the mutations were inherited from the patients’ parents (Figure S1). Ten Caucasian patients presented the *SLC2A9* variant c.374C>T; p.(T125M). Three of them were homozygous for this mutation, six were heterozygous, and one was a compound heterozygous with mutation c.374C>T; p.(T125M) in one allele and c.224T>G; p.(L75R) in the other allele. These two *SLC2A9* variants are very rare; p.(T125M) appears with a frequency of 7.75 × 10^−5^ in the European non-Finnish population (1.31 × 10^−4^ in total population) and p.(L75R) with a frequency of 4.03 × 10^−5^ in the European non-Finnish population (1.61 × 10^−5^ in total population) (data from gnomAD v2.1.1. Available online: https://gnomad.broadinstitute.org/).

**Figure 1:**
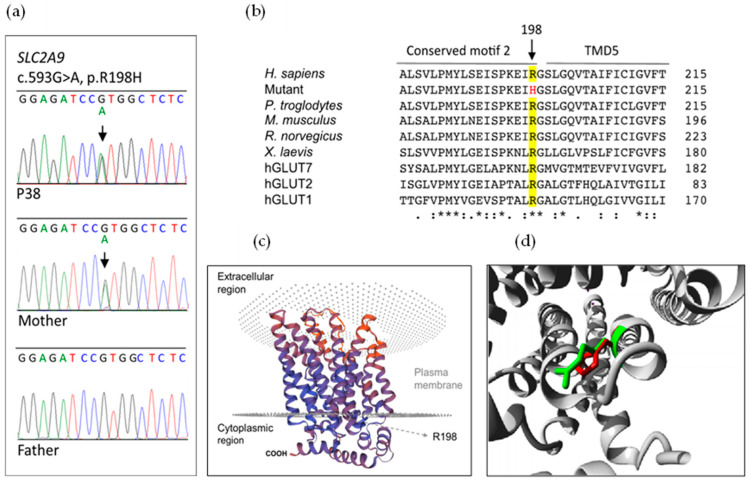
Identification of a new *SLC2A9* variant in patient P38 (family 29). (**a**) Electropherograms showing a partial sequence of *SLC2A9* exon 6 and the novel heterozygous *SLC2A9* variant c.593G>A detected in the patient and his mother. Vertical arrows indicate the position of the altered nucleotide. The G to A change results in amino acid substitution p.R198H in the encoded GLUT9 protein. Analysis of the patient’s father revealed the normal sequence. (**b**) Multiple alignments of partial human GLUT9 sequences with a subset of vertebrate orthologs and other GLUT family transporters. The vertical arrow indicates the position of the altered amino acid residue. Residues conserved at this position are highlighted in yellow. TMD5, Transmembrane domain 5. An asterisk indicates positions that have a single, fully conserved residue. A colon denotes conservation between groups of strongly similar properties. A period indicates conservation between groups of weakly similar properties. No symbol means no conservation. (**c**) Three-dimensional model for GLUT9 based on human GLUT1 crystal structure. The arrow indicates the position of arginine 198. (**d**) Close-up showing wild-type arginine 198 (green) and mutant histidine residue (red) associated with RHUC type 2.

**Figure 2:**
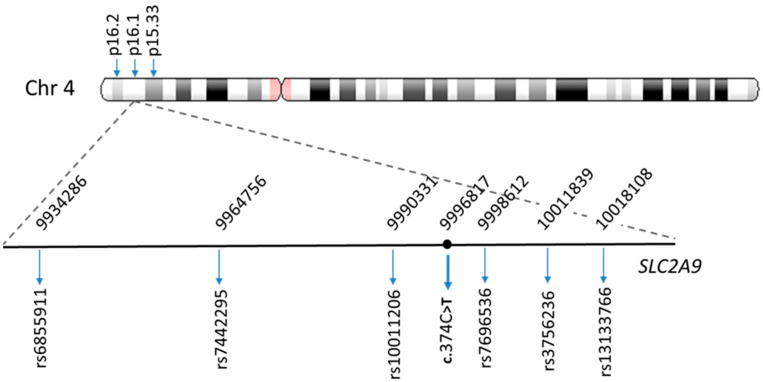
Location of the six *SLC2A9* TagSNPs used for haplotype analysis on the short arm of chromosome 4. The reference numbers of the SNPs are below the horizontal line. The numbers above the line indicate the position of each SNP on the chromosome (according to the Genome Variation Server 150). The position of variant c.374C>T is also indicated.

The other Caucasian patient (P38) was heterozygous for the *SLC2A9* variant c.593G>A; p.(R198H), identified in exon 5, which was not reported in the ClinVar or HGMD databases (Figure 1A). The amino acid numbering in the protein corresponds to the GLUT9L isoform (Ensembl transcript ID: ENST00000264784) (p.R169C in isoform GLUT9S). We submitted this novel *SLC2A9* variant to ClinVar and it was included with the accession number VCV001027407.1. In gnomAD version 2.1.1 (available online: https://gnomad.broadinstitute.org/) there were only three heterozygous alleles counted in the European (Non-Finnish) population, with an allele frequency of 2.33 × 10^−5^ (1.77 × 10^−5^ total population). Furthermore, no homozygotes have been reported. Variant c.593G>A was also present in a heterozygous state in the patient’s mother (Figure 1A). Amino acid residue R198 is located in the second cytoplasmic loop of GLUT9L between the fourth and fifth transmembrane segments and it is perfectly conserved among species and other GLUT family transporters (Figure 1B,C). The novel variant p.(R198H) is found at the conserved motif 2 (sugar transport proteins signature 2) (Figure 2B) [14]. The mutant histidine residue is smaller than the wild-type residue (Figure 1D). Additionally, the change in arginine for histidine results in a loss of positive charge.

According to Varsome (Available online: https://varsome.com/) the new *SLC2A9* variant, c.593G>A; p.(R198H), was considered as likely pathogenic using the criteria established by the American College of Medical Genetics and Genomics (1 strong, PM2, and 3 supporting, PM5, PP3, and PP5) [41]. It showed the pathogenic score employing 19 prediction tools and was benign with 2 tools.

### 2.3. Haplotype Construction of Patients with Recurrent SLC2A9 Variant c.374C>T

The results described above and the results of our previous studies showed that variant c.374C>T; p.(T125M) of *SLC2A9* is frequent among Spanish patients with RHUC type 2 (Table 1, [8,33]). To evaluate the hypothesis of a founder effect in patients with this recurrent mutation, a haplotype analysis was performed on 25 individuals. Some patients reported previously were included [8,33]. The TagSNPs selected (rs6855911, rs7442295, rs10011206, rs7696536, rs3756236, and rs13133766) for the haplotype analysis and their locations on chromosome 4 are shown in Figure 2. Major allele frequencies of the six selected markers are shown in Table S2 (Supplementary Materials).

Our results showed that alleles carrying the c.374C>T mutation shared a common SNP haplotype (Table 2). The distribution of this haplotype was significantly different between patients and controls (Table 2). Every patient harboring this mutation in a homozygous state had a common haplotype, G-A-C-T-G-T-C, (underlined, T is the c.374C>T mutant allele). This haplotype was present between RHUC patients at a 0.694 frequency. The haplotype G-G-C-C-G-T-C was the most frequent among controls (0.429). Thus, we concluded that mutation c.374C>T; p.(T125M) occurred due to a founder effect.

## 3. Discussion

RHUC type 1 and RHUC type 2 are rare inherited disorders caused by defective UA reabsorption in the renal proximal tubule due to mutations in the urate transporter URAT1 or GLUT9, respectively [11,14]. The regulation of these transporters is complex and takes place at different levels such as transcription and post-translation, and various signaling pathways are involved [42,43]. Recent results suggest that GLUT9 is regulated by insulin/insulin receptor signaling through PI3K/Akt activation, and by insulin-like growth factor-1 and its receptor through the activation of insulin receptor substrate 1, PI3/Akt, MEK/ERK, and p38 MAPK [44,45]. On the other hand, two PDZ domain-containing proteins PDZK1 and NHERF1 seem to regulate the transport activity of URAT1 via interaction with its PDZ motif located in the intracellular C-terminal region [46,47]. In spite of this progress, much remains to be learned about the regulation of urate transporters.

Our previous studies of RHUC with 13 Spanish families have identified mutations in these two genes [9,33]. Nine of these families were of Roma origin and presented the recurrent URAT1 (*SLC22A12*) pathogenic variants, mainly c.1400C>T; p.(T467M) and also c.1245_1253del; p(L415_G417del). The other four families were Caucasian and had shown the GLUT9 (*SLC2A9*) mutation, c.374C>T; p.(T125M), which had only been previously identified in an RHUC patient from Israel whose parents were both of Sephardi-Jewish origin living in Israel for many generations [25]. Transport and immunohistochemistry assays in *Xenopus* oocytes have revealed that mutation p.(T125M) significantly reduces the ability of GLUT9 to transport UA, and that this is due to a drastic decrease in the expression of the mutant protein [25,39].

The cohort analyzed in the present study included 21 patients from 18 new families. The genetic analysis showed that eight of these families, which are of Roma origin, had RHUC type 1, while the other ten had RHUC type 2. Of the 31 RHUC Spanish families studied so far, 13 (almost 42%) had the c.374C>T; p.(T125M) mutation in GLUT9. Therefore, we confirmed that this mutation is recurrent in Spanish families with RHUC type 2. This finding suggested the hypothesis of a founder effect. To evaluate this hypothesis, a set of six SNPs surrounding the position of variant c.374C>T; p.(T125M) were genotyped. The results showed that the Spanish patients and the patient of Sephardi-Jewish origin share a common c.374C>T-linked haplotype, which was not identified in the controls, indicating that this variant originated from a single mutational event. None of the RHUC type 2 patients who have been studied in other parts of the world carry this *SLC2A9* mutation [6,7,12,14,48], which excludes the hypothesis that c.374C>T; p.(T125M) originated from a mutation ‘hotspot’. Sephardi Jews are a Jewish migration population that arrived at the Iberian Peninsula mainly during the Roman Period [49]. By the late 15th century, Sephardi Jews were largely expelled from Spain, but many of them remained. Recent genetic studies have revealed a high level of Sephardic Jewish ancestry (approximately 20%) in the Spanish modern population (Iberian Peninsula and Balearic Islands) [50]. Therefore, we suggest that c.374C>T; p.(T125M) originated in the Sephardi population of Spain and spread there. It is also interesting to note that this variant is not present in the Ashkenazi population (Jews who settled in Central and Eastern Europe) (gnomAD v2.1.1. Available online: https://gnomad.broadinstitute.org/, accessed on 23 March 2023). On the other hand, based on the present findings and our previous results [9,33], c.374C>T; p.(T125M) accounts for the disease in up to 93% of the families with RHUC type 2. Since c.374C>T is very rare in the total population and the European non-Finnish population, the founder effect explains the high frequency of this mutation in the Spanish families. Some founder mutations are postulated to provide the carriers with a selective advantage; nevertheless, to our knowledge there is no obvious benefit for heterozygous carriers of *SLC2A9* mutations.

Our results also confirmed that the heterozygous loss-of-function GLUT9 mutation p.(T125M) can cause RHUC type 2 (Table 1). This has also been observed in two additional cases studied by our group [9]. Matsuo et al., were the first to identify cases of RHUC type 2 caused by two other heterozygous missense GLUT9 mutations, p.R380W and p.R198C [14]. Dinour et al., have suggested that heterozygous GLUT9 mutations cause mild hypouricemia most likely through haploinsufficiency [25]. As observed in Table 1, patients with the heterozygous p.(T125M) mutation have higher serum UA levels (1.0–1.9 mg/dL) and lower UA excretion (19–39%) compared to patients with the mutation in the homozygous state (0.12–1.20 mg/dL and 41–190%, respectively). Therefore, it seems that, in contrast to homozygous patients, heterozygous individuals maintain a certain ability to transport UA. Additionally, our clinical data showed that patients with the homozygous GLUT9 p.(T125M) mutation have a more severe hypouricemia (serum UA levels of 0.12–1.20 mg/dL; FE UA of 41–190%) than those with the homozygous URAT1 p.(T467M) mutation (serum UA levels of 0.70–1.40 mg/dL; FE UA of 22–57%) (Table 1). Other studies have reported similar results for the same GLUT9 and URAT1 mutations or for different mutations [4,6,9,24,25,48]. As has been suggested, this could be explained because UA reabsorption from the tubular lumen is facilitated by URAT1 as well as other apical UA transporters such as the organic anion transporters OAT4 and OAT10, while the UA exit to the blood is mediated solely by the basolateral GLUT9L [6,51]. Therefore, the complete loss of URAT1 activity would result in a partial UA reabsorption deficiency in patients with RHUC type 1, while the total loss of GLUT9 function would block UA reabsorption by all the apical transporters causing a total reabsorption defect in patients with RHUC type 2.

Patient 33, presenting the compound heterozygous mutations p.(T125M) and p.(L75R), showed serum UA levels (0.4 mg/mL) and UA excretions values (45%) similar to those patients with the p.(T125M) mutation in both alleles. This is consistent with previous findings reported for other compound heterozygous mutations [7]. The missense mutation p.(L75R) was first identified in several members of a consanguineous Israeli-Arab family [52]. Similarly to p.(T125M), the mutation c.224T>G; p.(L75R) is poorly expressed in the *Xenopus* expression system leading to a decreased UA transport activity [25,39]. This mutation is also very rare in the European non-Finnish population and in the total population.

Sequence analysis of *SLC2A9* in patient P38 (family 29) identified a very rare heterozygous missense mutation variant, c.593G>A; p.(R198H), which was inherited from his mother (Figure 2). This variant was considered as a likely pathogenic variant according to ACMG criteria using the Varsome database. Arginine 198 of GLUT9 is highly conserved among species and other GLUT family transporters, and it is found at the conserved motif 2 of GLUT family transporters (sugar transport proteins signature 2) located in the second intracellular loop region of the protein [14,53,54] (Figure 1B,C). It has been shown that arginine 153 in GLUT4, which corresponds to arginine 198 in GLUT9, is important for its proper conformation [53]. Matsuo et al., have previously reported a loss-of-function heterozygous GLUT9 mutation in the same codon, c.592C>T; p.(R198C) [14]. Expression experiments in *Xenopus* oocytes revealed that p.(R198C) abolishes urate transport activity [14,39]. This mutation also results in a loss of positive charge in the conserved motif 2 of GLUT9 [14]. All these findings indicate that the *SLC2A9* variant p.(R198H) is pathogenic and causes renal hypouricemia type 2 by decreasing urate reabsorption in the proximal tubule. Nevertheless, functional studies using the *Xenopus* oocyte expression system and electrophysiology will be required to characterize the consequences of this novel *SLC2A9* mutation.

In conclusion, our results indicate that the pathogenic variant c.374C>T; p.(T125M) of *SLC2A9* is the main cause of RHUC type 2 among Spanish patients and confirm that the *SLC22A12* variant c.1400C>T; p.(T467M) is the most frequent in patients of Roma origin. These data considerably facilitate the genetic diagnosis of RHUC in Spain. Furthermore, we have expanded the mutation spectrum of RHUC type 2 with the finding of a novel *SLC2A9* mutation. Finally, the haplotype analysis results confirmed that p.(T125M) is a founder mutation.

## 4. Patients and Methods

### 4.1. Patients

A total of 21 patients from different hospitals in Spain, 18 in pediatric age and 3 adults, belonging to 18 unrelated families diagnosed with idiopathic RHUC were investigated (Table 1). All patients were Spanish except P37 (family 28) and P38 (family 29) who were originally from South America, although of Spanish origin. Demographic data, medical history, clinical data, and biochemical data were obtained from all individuals. Ten affected subjects were Caucasians, ten were of Roma ancestry, and one (P32) was of unknown origin (the child was adopted). Blood and urine samples were collected for biochemical and genetic analysis. The group was composed of 9 females and 12 males, with ages ranging from 0.5 to 69 years. The criteria for the inclusion of patients were low levels of serum uric acid (sUA) (<2.0 mg/dL), high fractional excretion of uric acid (FE UA) (>10%), and other biochemical values normal. Serum levels of UA and creatinine, FE UA, and other biochemical values were determined using standard techniques. Subjects were evaluated for other renal diseases. This study was carried out following protocol PI 56-17, approved by the Ethics Committee of Hospital Universitario Nuestra Señora Candelaria, Santa Cruz de Tenerife, Spain with written informed consent from all subjects in accordance with the Declaration of Helsinki.

### 4.2. Mutation Analysis

The mutational analysis of *SLC22A12* and *SLC2A9* was performed using PCR amplification of the coding exons and direct DNA sequencing as previously described [9]. Genomic DNA of patients and relatives was extracted from peripheral blood samples using the GenElute Blood Genomic DNA kit (Sigma-Aldrich, St. Louis, MO, USA) following the manufacturer’s instructions. PCR products were purified with the NucleoSpin Gel and PCR Clean-up kit (Macherey-Nagel, Düren, Germany), and purified products were sent out to Macrogen Spain Inc. (Madrid, Spain) for DNA sequencing. DNA variants were identified by comparison to the respective reference *SLC22A12* (NCBI: NG_008110.1) and *SLC2A9* (NCBI: NG_011540.1) sequence and confirmed by sequencing additional independent amplification products.

Several databases of genetic variants including the Genome Aggregation Database (gnomAD. Available online: https://gnomad.broadinstitute.org/, accessed on 23 March 2023) [55], 1000 Genomes Project (available online: http://www.1000genomes.org/, accessed on 26 May 2022) [56], ClinVar (available online: https://www.ncbi.nlm.nih.gov/clinvar/, accessed on 26 May 2022) [57], and Human Gene Mutation Database (HGMD) (available online: http://www.hgmd.cf.ac.uk/ac/index.php, accessed on 7 March 2023) [58] were inquired for the presence and frequency of the new *SLC2A9* variant identified.

### 4.3. In Silico Analysis of Variants

The pathogenicity of the novel *SLC2A9* variant was evaluated using Varsome, an annotation tool and search engine for human genomic variants which estimates the impact of mutations on protein structure and function (available online: https://varsome.com/, accessed on 26 August 2022) [59]. With this tool, variant pathogenicity is reported using an automatic classifier that evaluates the submitted variant according to the American College of Medical Genetics and Genomics (ACMG) guidelines [41]. The variant of interest is classified as pathogenic, likely pathogenic, benign, likely benign, or uncertain significance.

The multiple sequence alignment of proteins was performed using Clustal Omega (1.2.4) (available online: https://www.ebi.ac.uk/Tools/msa/clustalo/, accessed on 10 October 2022) [60]. Protein sequences were obtained from the National Center for Biotechnology. Accession numbers: human GLUT9, NP_064425.2; *Pan troglodytes* GLUT9, XP_016806818.1; *Mus musculus* GLUT9, NP_001095884.1; *Xenopus laevis* GLUT9, NP_001121314.1; human GLUT7, NP_997303.2; human GLUT2, NP_000331.1; and human GLUT1, NP_006507.2.

### 4.4. Protein Modeling

We used the protein structure homology-modeling server SWISS-MODEL (available online: https://swissmodel.expasy.org/, accessed 16 November 2022) to generate the three-dimensional structure (3D) of wild-type GLUT9 using GLUT1 protein as a template. The web service HOPE (available online: https://www3.cmbi.umcn.nl/hope/, accessed on 9 November 2022) was used to analyze the structural effects of the novel missense mutation in GLUT9.

### 4.5. Haplotype Analysis

In order to test whether the patients had a common c.374C>T; p.(T125M)-linked haplotype, an SNPs-haplotype analysis was performed. In this analysis, we also included a DNA sample from the Israeli RHUC patient of Sephardi-Jewish origin in whom the mutation was originally identified (kindly provided by Drs. Dganit Dinour and Liat Ganon) [25]. Six informative intronic SNPs (dataset CEU population) (CEU means Northern Europeans from Utah. Utah residents with Northern and Western European ancestry) from separate LD bins flanking the c.374C>T variant site were selected using the Genome Variation Server (GVS. Available online: https://gvs.gs.washington.edu/GVS150/index.jsp, accessed on 2 March 2022) with the criterion of R2 threshold 0.8. The SNPs were analyzed using PCR amplification followed by DNA sequencing. Primer3 software was used to design the specific oligonucleotide primers for all fragments to be amplified that included the selected SNPs (Table S1, Supplementary Materials). The amplification products were analyzed by Sanger sequencing (Macrogen Inc., Madrid, Spain). Patients’ haplotypes were reconstructed by inspection of all SNPs. Allelic and haplotypic frequencies were determined using GENEPOP 4.7 (available online: http://kimura.univ-montp2.fr/~rousset/Genepop.htm, accessed on 2 March 2022), PHASE v2.1.1 (available online: http://stephenslab.uchicago.edu/phase/download.html, accessed on 2 March 2022), and SNPSTATS (available online: https://www.snpstats.net/start.htm, accessed on 2 March 2022). The Hardy–Weinberg Equilibrium was verified for all the SNPs.

## Figures and Tables

**Table 1:** Characteristics of patients with renal hypouricemia.

Patient/ Family	Gender	Age (Years)	SUA (mg/mL)	FE UA (%)	SCrea (mg/dL)	Renal Symptoms	Ethnicity	*SLC22A12* Mutation	*SLC2A9* Mutation
P20/F14	M	4	1.70	26	0.34	-	Caucasic	-	p.T125M
								-	-
P21/F15	F	13	0.12	190	0.49	-	Caucasic	-	p.T125M p.T125M
								-	
p.T125M	M	16	0.14	170	0.54	-	Caucasic	-	
								-	
p.T125M	F	18	0.80	22	0.50	UTI	Roma	p.T467M	-
								p.T467M	-
P24/F16	F	43	0.90	37	0.66	NL	Roma	p.T467M	-
								p.T467M	-
P25/F17	F	6	1.90	19	0.60	-	Caucasic	-	p.T125M
								-	-
P26/F18	M	4	1.10	28	0.29	-	Roma	p.T467M	-
								p.T467M	-
P27/F19	M	13	1.40	38	0.86	NS	Roma	p.T467M	-
								p.T467M	-
P28/F19	M	3	1.30	37	0.39	-	Roma	p.T467M	-
								p.T467M	-
P29/F20	F	69	0.90	41	0.84	-	Caucasic	-	p.T125M
								-	p.T125M
P30/F21	F	55	1.2 *	48 *	0.80 *	RPGN	Roma	p.T467M	-
								p.T467M	-
P31/F22	M	18	1.00 **	61 #	0.77 **	EIARF	Roma	p.T467M	-
								p.L415_G417del	-
P32/F23	M	8	1.10	22	0.35	-	Unknown	-	p.T125M
								-	-
P33/F24	M	7	0.4	45	0.39	Renal scar	Caucasic	-	p.T125M
								-	p.L75R
P34/F25	F	0.5	1.56	24	0.22	-	Caucasic	-	p.T125M
								-	-
P35/F26	M	5	0.95	25	0.35	UF	Roma	p.T467M	-
								p.T467M	-
P36/F27	M	7	1.10	41	0.35	-	Roma	p.T467M	-
								p.T467M	-
P37/F28	F	5	1.00	39	0.47	-	Caucasic	-	p.T125M
								-	-
P38/F29	M	2	1.10	46	0.29	-	Caucasic	-	p.R198H
								-	-
P39/F30	F	11	0.70	57	0.49	-	Roma	p.T467M	-
								p.T467M	-
P40/F31	M	8	1.40	28	0.45	UF	Caucasic	-	p.T125M
								-	-

SUA: Serum Uric Acid. FE UA: Fractional Excretion of UA. SCrea: Serum Creatinine. M: Male. F: Female. UTI: Urinary Tract Infection. NL: Nephrolithiasis. NS: Nephrotic Syndrome. RPGN: Rapid Progressive Glomerulonephritis. EIARF: Exercise-Induced Acute Renal Failure. UF: Urinary Frequency. * Data obtained after complete remission of RPGN (with nephrotic proteinuria and hematuria). ** Data obtained before the EIARF. # Data obtained during the hospitalization of the patient (three days after the acute kidney failure). Reference values: Serum UA, children under 15 and adult females, 2.0 to 5.7 mg/dL; adult males, 2.0 to 7.0 mg/dL. FE UA, children under 15 years and adult females, 7.3 ± 1.3%; adult males, 10.3 ± 4.2%. Serum creatinine, 0.5 to 1.2 mg/dL.

**Table 2:** Inferred haplotypes. Patients carrying the c.374C>T mutation share a common SNP haplotype, shown in shaded boxes.

	SNP1_ 9934286	SNP2_ 9964756	SNP3_ 9990331	Mutation c.374C>T_ 9996817	SNP4_ 9998612	SNP5_ 10011839	SNP6_ 10018108	Total (%)	Controls (%)	Patients (%)
1	G	A	C	**T**	G	T	C	0.5	NA	0.694
2	G	G	C	C	G	T	C	0.2	0.429	0.111
3	A	A	T	C	T	T	C	0.14	0.286	0.083
4	A	A	C	C	G	A	T	0.08	0.214	0.009
5	G	A	C	C	G	A	T	0.04	NA	0.056
6	G	G	C	T	G	T	C	0.02	NA	0.028
7	G	G	C	C	G	A	T	0.02	0.071	NA

## Data Availability

The data presented in this study are available on request from the corresponding author.

## Supplementary Materials

The following supporting information can be downloaded at: https://www.mdpi.com/article/10.3390/ijms24098455/s1.
