# Supplementary material for: Pathogenic Variants of SLC22A12 (URAT1) and SLC2A9 (GLUT9) in Spanish Patients with Renal Hypouricemia: Founder Effect of SLC2A9 Variant c.374C>T; p.(T125M)
Source: Int J Mol Sci. 2023 May 8;24(9):8455. doi: 10.3390/ijms24098455 (PMC10179447; doi:10.3390/ijms24098455)
Supplement: Supplementary file 1 [file ijms-24-08455-s001.zip › ijms-2365849-supplementary.pdf]

Supplementary material

(a)

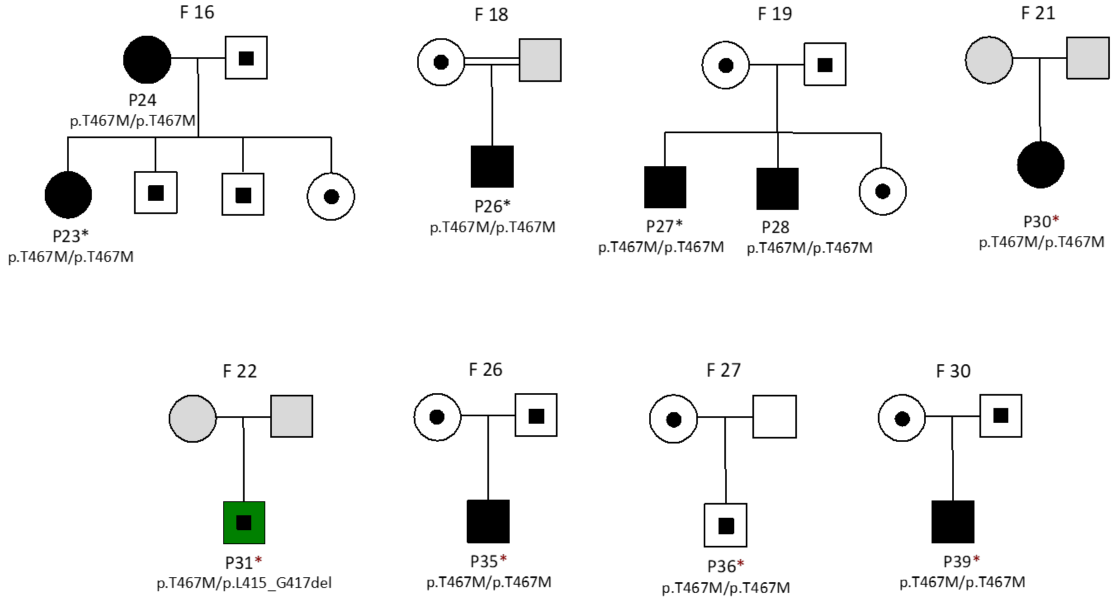

(b)

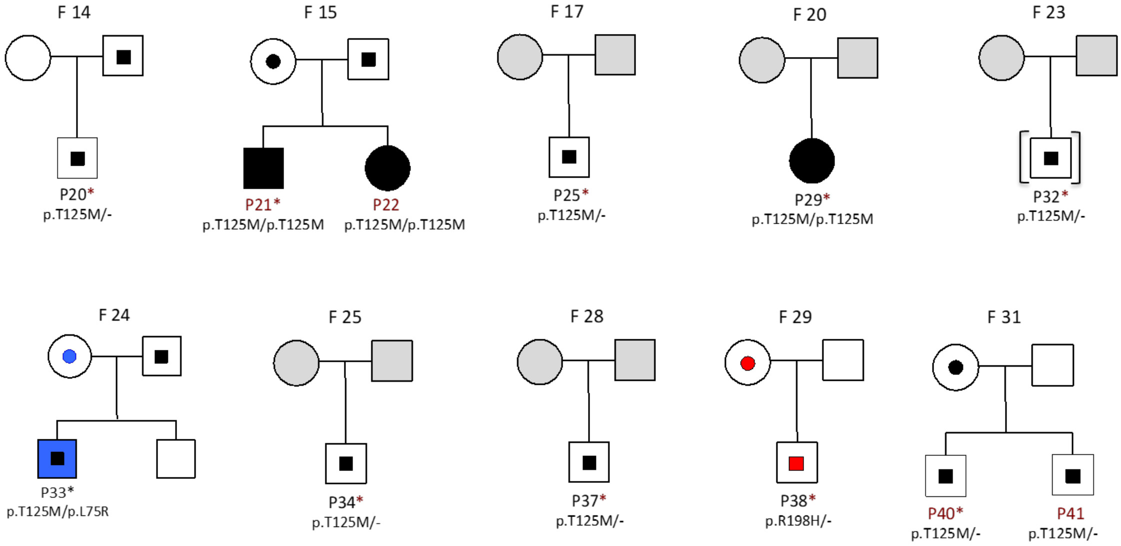

Figure S1. Pedigrees of families with RHUC type 1 **(a)** and type 2 **(b)** showing segregation of *SLC22A12* and *SLC2A9* variants, respectively. Asterisks indicate probands in each pedigree. Circles and squares correspond to female and male individuals, respectively. Filled circles and squares represent homozygous or compound heterozygous subjects; filled circles and squares within open frames represent heterozygous individuals; unfilled symbols denote subjects without mutation; grey symbols represent family members who were not available for genetic analysis. Double line indicates paternal consanguinity. F, family. Brackets indicates adoption. Black symbols indicate subjects harbouring the most frequent mutation in RHUC type 1 or RHUC type 2. Green symbols indicate individual

carrying mutation p.(L415_G417del). Blue symbols indicate individuals carrying mutation p.L75R. red symbols represent individuals harbouring novel mutation p.R198H.

Table S1: Primers used for PCR amplification of the variants genotyped for haplotype analysis. Human assembly GRCh38.p13

	**Reference SNP**	**Position**	**Primers sequences (5′ – 3′)**
SNP_1	rs6855911	chr4:9934286	rs6855911F: CTCCCAATCAAATCCCCTCT<br>rs6855911R: TGAAAAGCCATCTGTGGTCA
SNP_2	rs7442295	chr4:9964756	rs7442295F: GGCTGGGGCTTAAAATCACT<br>rs7442295R: GAGAGGCAAGAGGGCTGTC
SNP_3	rs10011206	chr4:9990331	rs10011206F: ATGAGGGAACGGAAAGTGTG<br>rs10011206R: GCCCTTCAACGTAAGAACCA
SNP_4	rs7696536	chr4:9998612	rs7696536F: GCAGTGTGTTGTCTGCTGGT<br>rs7696536R: GGCTGTTTCTCGTCTGGAGT
SNP_5	rs3756236	chr4:10011839	rs3756236F: GCCAATTGGAGCTAGTCACTG<br>rs3756236R: GAAAAGGGAGGAGGCTCTTG
SNP_6	rs13133766	chr4:10018108	rs13133766F: CATCCATCACTCCACCTTCC<br>rs13133766R: CACATTGTGCCTCTGGTTTG

Table S2. Allele frequencies distribution of the selected SNPs markers in cases and controls.

	Alelle	Controls (%)	RH patients (%)
SNP_1 G/A	G	0.5	0.89
SNP_2 A/G	A	0.5	0.86
SNP_3 C/T	C	0.75	0.92
SNP_4 G/T	G	0.75	0.92
SNP_5 T/A	T	0.67	0.94
SNP_6 C/T	C	0.71	0.94
